# 

**Published:** 1916-12-23

**Authors:** 


### CONTENTS.

PAGE

**Industrial Efficiency and Eyesight** ... ... 235

**An Avoided Danger to Voluntary Hospitals...** 236

**Hospital and Institutional News ...** ... ... 237

Death of Sir Frederic Eve—New Secretary to the Middlesex Hospital—Handsome New Annual Subscriptions—A New Appointment—Presentation to a Hospital Chairman—Charges Against a Walsall Practitioner Upheld—Women Residents at the London Hospital—Doctors' Salaries in Ireland—Officers as Out-Patients—Secret Critics of the Welsh Memorial Association—Mr. W. Crooks as an In-Patient—A Big Hospital for German Prisoners—The Baker Street Bazaar — Trinitrotoluene Poisoning — Treatment — The Cocaine and Opium Regulations and Their Effect on Institutions—A Unique Chapel—This Week's Drug Market.

**Sleeplessness** ... ... ... ... ... 241

A Difficult Problem in Therapeutics.

**The Triumph of the Voluntary Principle** ... 243

By the Rev. R. J. Campbell, M.A.

**King Edward's Hospital Fund for London** ... 245

Distribution Meeting of the Governors and General Council: Increase in Awards of £30,000.

Report of the Distribution Committee.

Grants to Hospitals Recommended by the Distribution Committee.

Report of the Convalescent Homes Committee.

Grants Recommended by the Convalescent Homes Committee.

**The Matrons' and Sisters' Department** ... 251

Our Women, Warriors, and Workers.

Round the Hospitals.

Professions Favoured by Graduates—The Teaching of Dietetics—How to Train for an Examination —Training in America—Poor-Law Officers and Trained Nurses.

**The League of Mercy...** ... ... ... ... 253

Annual Meeting at St. James's Palace.

**Women in Medicine. 1866—1916** ... ... 254

New Hospital for Women.

**The Editor's Letter-Box** ... ... ... ... iv

Lord Roberts's Field Glasses: A Retrospect of Two Years' Work.

**The Institutional Worker** ... ... (Suppl.) 1

The Origin and Growth of a Workpeople's Fund.

Question for December.

Questions Invited.

Enquiries and Answers.

